# Regulator of Calcineurin 1 in Periodontal Disease

**DOI:** 10.1155/2016/5475821

**Published:** 2016-06-15

**Authors:** Ulrike Peters, Eleni Solominidou, Yüksel Korkmaz, Stefan Rüttermann, Astrid Klocke, Thomas Frank Flemmig, Thomas Beikler

**Affiliations:** ^1^Section of Periodontics, Heinrich-Heine University, 40225 Düsseldorf, Germany; ^2^Institute for Experimental Dental Research and Oral Musculoskeletal Biology, Center for Biochemistry, University of Cologne, 50931 Cologne, Germany; ^3^Department of Operative Dentistry, Center for Dentistry and Oral Medicine (Carolinum), Goethe-University Frankfurt, 60596 Frankfurt, Germany; ^4^Faculty of Dentistry, The University of Hong Kong, Sai Ying Pun, Hong Kong

## Abstract

Nuclear factor of activated T-cells (NFAT) and NF-kB pathway associated processes are involved in the pathogenesis of various inflammatory disorders, for example, periodontal disease. The activation of these pathways is controlled by the regulator of calcineurin 1 (RCAN1). The aim of this study was to elucidate the role of RCAN1 in periodontal disease. Healthy and inflamed periodontal tissues were analyzed by immunohistochemistry and immunofluorescence using specific rabbit polyclonal anti-RCAN1 antibodies. For expression analysis human umbilical vein endothelial cells (HUVEC) were used. HUVEC were incubated for 2 h with Vascular Endothelial Growth Factor (VEGF) or with wild type and laboratory strains of* Porphyromonas gingivalis* (*P. gingivalis*). Expression analysis of* rcan1* and* cox2* was done by real time PCR using specific primers for* rcan1.4* and* cox2*. The expression of* rcan1* was found to be significantly suppressed in endothelial cells of chronically inflamed periodontal tissues compared to healthy controls.* Rcan1* and* cox2* were significantly induced by VEGF and wild type and laboratory* P. gingivalis* strains. Interestingly, the magnitude of the* rcan1* and* cox2* induction was strain dependent. The results of this study indicate that RCAN1 is suppressed in endothelial cells of chronically inflamed periodontal tissues. During an acute infection, however,* rcan1* seems to be upregulated in endothelial cells, indicating a modulating role in immune homeostasis of periodontal tissues.

## 1. Introduction

Periodontitis is a chronic inflammatory disease resulting in the destruction of periodontal tissues and, if left untreated, in tooth loss. It is well accepted that dysbiotic microbial communities within the oral cavity are involved in the onset and progression of periodontal diseases [1, 2]. These communities display synergistic virulence that can evade the host immune response and trigger tissue-destructive inflammatory and immune responses [3]. Many of these processes are under control of the nuclear factor of activated T-cells (NFAT) [4, 5] and the NF-kB pathway [6–10]. NFAT activation induces the expression of various cytokines, including IL-2, IL-3, IL-4, IL-5, IL-6, TNF-*α*, and GM-CSF, whereas induction of NF-kB results in an increased expression of inflammatory cytokines like IL-1b, IL-6, TNF-*α*, and IL-8 [9].

The regulator of calcineurin 1 (RCAN1) regulates NFAT and NF-kB depending pathways. The human RCAN1 genes are expressed as two isoforms, that is, RCAN1.1 and RCAN1.4 [11, 12]. While RCAN1.1 seems to be constitutively expressed in most tissues, transcription of the RCAN1.4 variant is induced de novo by several stimuli [13]. RCAN1 interacts with cytosolic calcineurin to inhibit its phosphatase activity and thus the dephosphorylation, nuclear translocation, and activation of NFAT [5, 14, 15]. In addition to that RCAN1 led to enhanced stability of a family of NF-kB inhibitory molecules thus suppressing the NF-kB pathway [9, 16]. RCAN1 is highly expressed in various tissues of the human body, including heart, lung, kidney, brain, muscle, liver, and testis [9]. Furthermore, it has been implicated in important physiological and pathological processes, including cell growth and immune regulation [13].

The human* rcan1* gene is located within the Down Syndrome critical region on chromosome 21 and is overexpressed in individuals with trisomy 21 [17]. This overexpression has been implicated to mediate some of the infectious complications associated with this syndrome [9, 17]. In this regard it is noteworthy that severe periodontitis is a common manifestation among subjects with Down Syndrome, with an estimated prevalence of 58–96% in those under 35 years of age [18]. Moreover, the expression of* rcan1* has also been found to be upregulated in periodontal tissues following mechanical stress and nonsurgical periodontal therapy [19, 20], indicating a role in homoeostasis of periodontal tissues.

Taken together these findings suggest that RCAN1 is involved in the pathogenesis of periodontal diseases. Therefore, the aim of the present study was to further assess the potential role of RCAN1 in periodontal tissues by histological and coincubation studies.

## 2. Materials and Methods 

### 2.1. Tissue Samples Collection and Tissue Preparation

Healthy (*n* = 6) and (*n* = 6) third molars with chronic periodontitis that had to be extracted for orthodontic/medical reasons were included in the study. The patients agreed to have the tissue biopsies taken and examined for research purposes. Procurement of human teeth tissues at surgery was approved by the “Ethikkommission an der Medizinischen Fakultät der Heinrich-Heine-Universität Düsseldorf” (institutional review board of the Heinrich-Heine-University Düsseldorf; IRB approval number 2980). The patients agreed to have the extracted teeth examined for research purposes. The molars and the adherent periodontal ligament (PDL) were immersion-fixed in a fixative (4% paraformaldehyde and 0.2% picric acid in 0.1 M phosphate buffer saline (PBS), pH 7.4) and demineralized for 21 days in 4 N formic acid. The samples were cryoprotected, frozen embedded, and frozen-sectioned on a cryostat at 30 *μ*m sections. To determine the health and inflammation state of the teeth, sections were characterized by Haematoxylin and Eosin (H&E) staining as previously described [21].

### 2.2. Immunohistochemistry

#### 2.2.1. Avidin-Biotin-Peroxidase Staining

Sections of healthy and inflamed molars were incubated for 48 h at 4°C with rabbit polyclonal anti-RCAN1. Then, sections were incubated with biotin-conjugated goat anti-rabbit IgG (1 : 500) and followed by avidin-biotin-peroxidase complex (Vector, Burlingame, CA; 1 : 100). The signal was visualized with 0.05% 3,3′-diaminobenzidine tetrahydrochloride (Sigma-Aldrich, Taufkirchen, Germany). Incubations without the primary antisera were carried out as immunohistochemical controls [21].

#### 2.2.2. Double-Immunofluorescence and Confocal Microscopy

The free-floating sections were incubated with endothelial cell marker mouse anti-CD31 (1 : 800) for 24 hrs at 4°C. Then, sections were incubated at 1 : 500 dilution with DyLight*™* 488-conjugated goat anti-mouse IgG (Pierce Biotechnol., Rockford, IL) for 1 h at RT and with rabbit anti-RCAN1.4 (Santa Cruz, Heidelberg, Germany) for 24 hrs at 4°C. Thereafter, the sections were incubated with DyLight 549-conjugated goat anti-rabbit IgG (Pierce; 1 : 500) for 1 h at RT. The nuclei were stained for 15 min with the DNA stain DRAQ5 (Axxora, Lörrach, Germany) at RT. The control sections were incubated without mouse anti-CD31 and rabbit anti-RCAN1.4 but with all reagents used in the immunohistochemical incubations [21]. Three colour fluorescent images were acquired on LSM 510 META confocal microscope (Carl Zeiss, Jena, Germany). The confocal images through regions of interest in each preparation at 0.1 *μ*m intervals throughout the depth of the section were collected for each fluorochrome at each z-step as described previously [21].

#### 2.2.3. Densitometry of the Immunohistochemical Stainings

The densitometric analysis was performed with a Zeiss Axioskop-2 Plus microscope at 100x magnification coupled with Image System Analysis, Axiovision Ver. 4.7 (Carl Zeiss, Jena, Germany). G-intensities of RCAN1 in endothelium of blood vessels of the healthy and inflamed periodontal ligament were measured by grey values of immunostaining. The background grey value was measured from three selected regions at a section-free area. The blood vessels grey values were measured from three selected areas of the blood vessels. Immunostaining intensity was presented as the mean of measured blood vessels grey value minus mean of measured background grey value [22].

### 2.3. Cell Culture

Human umbilical vein endothelial cells (HUVEC) were purchased (Promocell, Heidelberg, Germany) and grown in Endothelial Cell Growth Medium (Promocell, Heidelberg, Germany) at 37°C in a humidified atmosphere of 95% air and 5% CO_2_ in 75 cm^2^ flask until 70–90% confluence. The cells were harvested and seeded in tissue culture 6-well plates (Sarstedt, Germany) with counts of 60.000 cells/plate and used for experiments when having reached 80% confluence. Cells were used for experiments between passages 3 and 6. Cell viability was determined by Trypan Blue exclusion test.

### 2.4. Bacterial Culture

Wild type* Porphyromonas gingivalis (P. gingivalis)* were isolated from patients with chronic periodontitis. Type strain* P. gingivalis* DSM 20709 was obtained from the German Collection of Microorganisms and Cell Cultures Inc. (DSMZ Braunschweig, Germany). All bacterial strains were grown in liquid media containing 10% FCS, 3% TSB, 0.5% yeast, 0.05% L-cystein, 0.0005% hemin, and 0.001% vitamin K1 (all from Merck, Germany), in an anaerobic chamber (Meintrup, Germany) at an atmosphere of 80% N_2_, 10% H_2_, and 10% CO_2_. All stocks were grown to OD 0.5, centrifuged, and resuspended in an equal volume of Endothelial Cell Growth Medium (Promocell, Heidelberg, Germany).

### 2.5. RCAN1 and COX2 Expression Assays

HUVEC in 6-well plates grown to 80% confluence were incubated with 1 mL bacterial suspension OD 0.5 or with 25 ng/mL Vascular Endothelial Growth Factor (VEGF, Invitrogen Inc., Carlsbad, USA) or medium only for control. All plates were incubated for 2 h at 37°C in a humidified atmosphere of 95% air and 5% CO_2_. Following incubation cells were washed with Hepes-BSS, detached with Detach Kit (Promocell, Heidelberg, Germany) and total RNA was isolated with Qiagen Qiashredder and Qiagen RNeasy Mini Kit according to manufacturer's instructions (Qiagen, Germany). Quantity and quality check of RNA was performed with Agilent Bioanalyzer. All experiments were run in triplicate.

### 2.6. Real Time PCR

Complementary DNA was synthesized using iScript cDNA Synthesis Kit (Bio-Rad, Germany). Quantitative real time PCR was performed with 2.5 ng/*μ*L cDNA for* gapdh* and 10 ng/*μ*L cDNA for* rcan1* in a Bio-Rad CFX-96 Real Time System using SsoFast Eva Green Supermix (Bio-Rad, Germany). The qRT-PCR conditions were 95°C for 3 min followed by 40 cycles of 95°C for 10 s, 55°C for 5 s, 60°C for 10 s, and 77°C for 1 s. To verify that a specific product was amplified, a melting curve was generated at the end of PCR. Gene specific primers used were GAPDH f114 (5′-GAGTCAACGGATTTGGTCGT-3′), GAPDH r260 (5′-GACAAGCTTCCCGTTCTCAG-3′), and RCAN1 3r99 (5′-GCTCTTAAAATACTGAAAGGTG-3′) according to Yao and Duh [23], and RCAN 3F (5′-TGACTGCGTGGGTCTGTAGCGC-3′), COX2 exF1 (5′-GCCTGGTCTGATGATGTATG-3′), and exR (5′-GGGTAATTCCATGTTCCAGC-3′) designed for the present study and specific for RCAN1.4 and COX2, respectively. PCR products were verified by sequencing. Gene expression level relative to* gapdh* and control was calculated as ΔΔCq with the Bio-Rad CFX Manager 2.0 software (Bio-Rad, Germany).

### 2.7. Statistical Analysis

The statistical comparisons of the densitometric measurements between healthy and inflamed tissues were performed using Kruskal-Wallis test followed by two-tailed Student's *t*-test for paired samples. Differences in the expression pattern between HUVEC control and HUVEC test groups and between* rcan1* and* cox2* were analyzed by two-tailed Student's *t*-test for unpaired samples (SPSS, v.18, Munich, Germany). The significance level was set at *p* < 0.05.

## 3. Results

### 3.1. Localization of RCAN1 in Healthy and Chronically Inflamed Periodontal Ligament (PDL)

**Figure 1:**
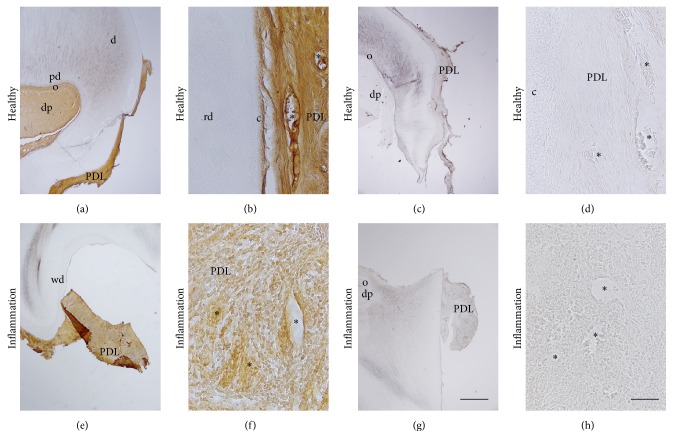
Localization of RCAN1 in healthy and chronically inflamed periodontal ligament (PDL). d = dentin, pd = predentin, o = odontoblasts, dp = dental pulp, rd = reactive dentin, PDL = periodontal ligament, and c = cementum. Asterisks indicate blood vessels (b) or negative erythrocytes indicating blocked endogenous peroxidase in blood vessels (d). Bars: (a, c, e, g) 1 mm; (b, d, f, h) 50 *μ*m.

RCAN1 was detected in blood vessels of the healthy PDL (Figure 1(a)). In higher magnification of the overview picture, blood vessels (asterisks) were stained by RCAN1 (Figure 1(b)). After incubation without the primary antibodies, secondary antibodies revealed no staining (Figure 1(c)). In higher magnification of c, negative erythrocytes (asterisks) indicated blocked endogenous peroxidase in blood vessels of the PDL (Figure 1(d)).

In inflamed PDL, staining intensity for RCAN1 was lower in blood vessels (asterisks) when compared with healthy PDL (Figures 1(e) and 1(f)). Numerous inflammatory cells were found in the PDL (Figure 1(f)). Incubation without the primary antibodies but with secondary antibodies only revealed no staining in the inflamed control sections (Figures 1(g) and 1(h)). In inflamed PDL, negative erythrocytes (asterisks) indicated blocked endogenous peroxidase in blood vessels (Figure 1(h)).

Densitometric analysis confirmed the significantly lower staining intensity for RCAN1 in endothelial cells of the inflamed (863.23 ± 49.60 densitometric unit) compared to endothelial cells of the healthy PDL (1422.42 ± 90.65 densitometric unit) (Figure 3).

**Figure 2:**
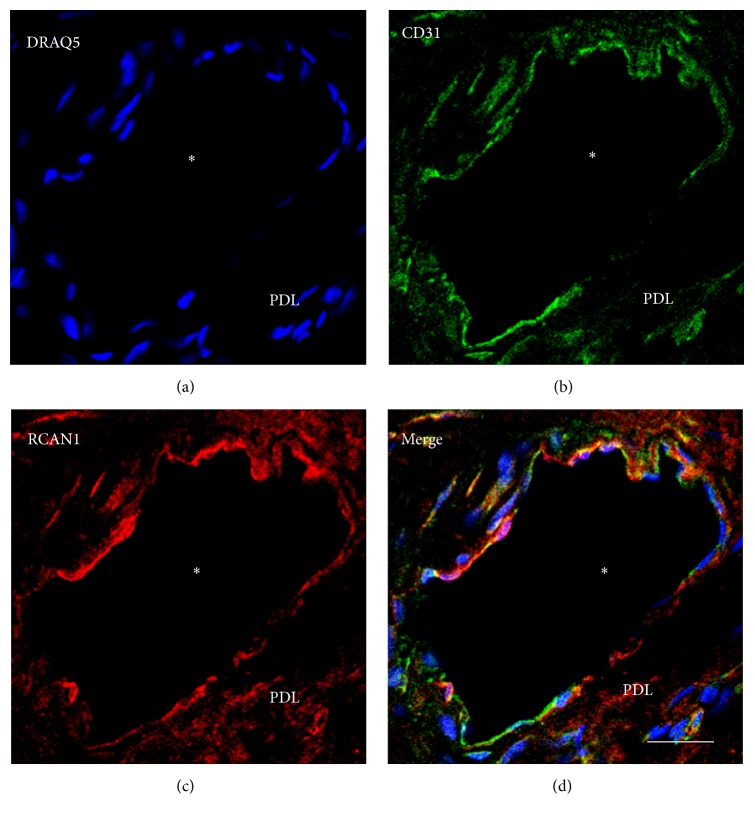
Confocal microscopy and immunofluorescence of RCAN1 in PDL. Cell nuclei of the PDL cells were identified by DRAQ5 (a; blue). The colocalization of CD31 (b: green; a marker for endothelial cells) and RCAN1 (c; red) in blood vessel walls of healthy PDL indicates that RCAN1 is localized in blood vessel endothelia. *∗* = lumen of blood vessel (d; merge; yellow); white bar = 20 *μ*m.

**Figure 3:**
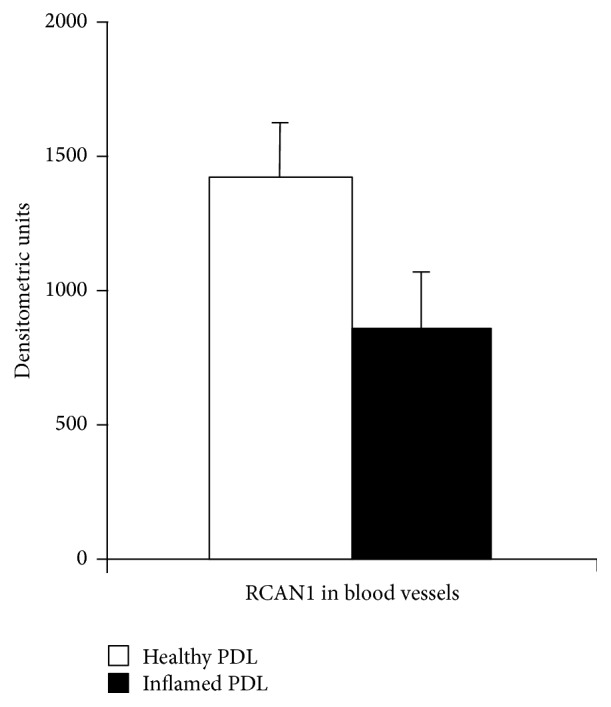
Densitometric analysis of RCAN1 in endothelial cells. Staining intensities (densitometric units) for RCAN1 in healthy and inflamed endothelial cells. Data are mean ± SD.

Confocal microscopy revealed expression of RCAN1 in endothelial cells of PDL by colocalization of CD31 and RCAN1. No colocalization with DRAQ5 and RCAN1 could be detected, indicating that RCAN1 is typically not detectable in the nuclei of PDL cells (Figures 2(a)–2(d)).

### 3.2. Expression of* Rcan1* and* Cox2* in Endothelial (HUVEC) Cells

**Figure 4:**
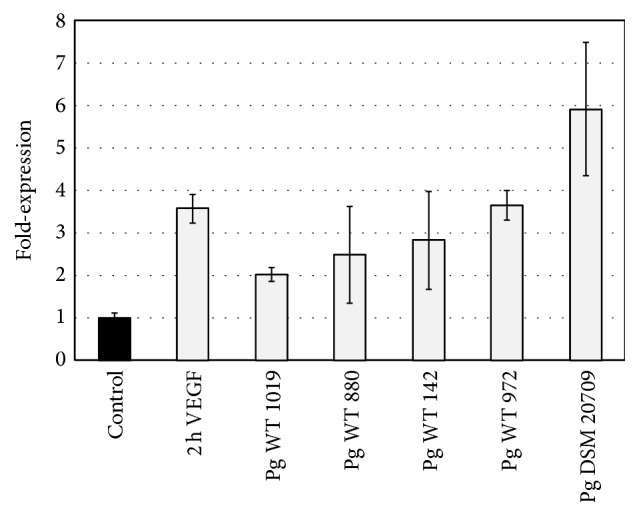
Expression of* rcan1* in endothelial (HUVEC) cells. Expression of* rcan1* following 2 h incubation with medium (negative control), VEGF (positive control), and* P. gingivalis* strains DSM 20709, WT 149, WT 880, WT 972, and WT 1019. Error bars indicate ±SD.

The incubation with VEGF for 2 h resulted in a significantly (*p* < 0.05) 3.56 ± 0.33-fold increased expression of* rcan1* compared to the untreated control (see Figure 4). In addition, wild type* P. gingivalis* significantly (*p* < 0.05) increased the expression of* rcan1 *2.02 ± 0.16 to 3.64 ± 0.35-fold compared to untreated controls. Incubation with the laboratory strain DSM 20709 induced a 5.9 ± 1.56-fold increase in expression compared to untreated controls. The increase was found to be significantly (*p* < 0.05) increased compared to expression of* rcan1* induced by wild type strains of* P. gingivalis* as well as to the untreated control.

**Figure 5:**
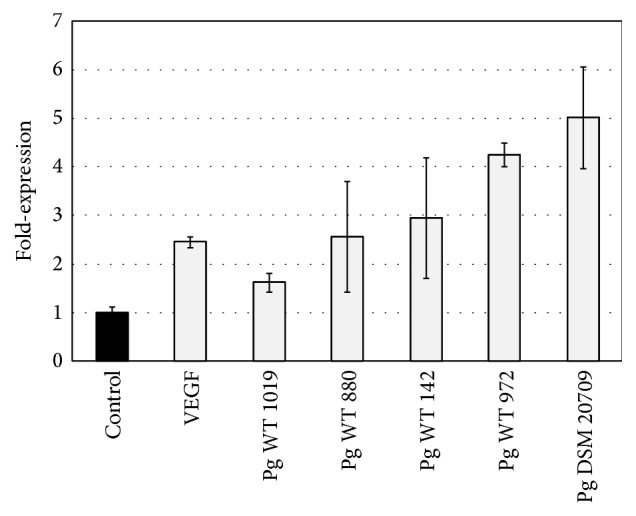
Expression of* cox2* in endothelial (HUVEC) cells. Expression of* cox2* following 2 h incubation with medium (negative control), VEGF (positive control), and* P. gingivalis* strains DSM 20709, WT 149, WT 880, WT 972, and WT 1019. Error bars indicate ±SD.

VEGF induced a significant 2.5 ± 0.1-fold increased (*p* < 0.05) expression of* cox2* compared to the untreated control (see Figure 5). The VEGF induced expression of* cox2* was found to be significantly lower than the VEGF induced expression of* rcan1.*

Wild type* P. gingivalis* significantly (*p* < 0.05) increased the expression of* cox2* in a range from 1.65 ± 0.15- to 4.32 ± 0.18-fold compared to the untreated controls. The* cox2* expression pattern induced by wild type* P. gingivalis* strains was found to be similar and not statistically different from the* rcan1* expression.

Incubation with laboratory strain DSM 20709 resulted in a 5.01 ± 1.05-fold increased expression of* cox2* compared to untreated controls. This increase was found to be significantly (*p* < 0.05) increased compared to the* cox2* induced expression by wild type strains of* P. gingivalis* as well as to the untreated control. Compared to the* rcan1* expression, however, the* P. gingivalis* DSM 20709 induced* cox2 *expression was found to be significantly lower.

## 4. Discussion

To our knowledge, this is the first reported histological evidence of RCAN1 expression in endothelial cells of periodontal tissues.* Rcan1* is a VEGF target gene in endothelial cells that regulates NFAT [5, 14, 15] and NF-kB dependent pathways [9, 16] as well as other inflammatory mediators like COX2, PGE_2_, and thromboxane [10, 15]. These are signaling pathways and molecules that regulate the inflammatory response in periodontal tissues.

RCAN1 has been found as both an inhibitor [24–26] and an activator [27–29] of inflammation. The apparently paradoxical actions of RCAN1 can be attributed to RCAN1's unique function in a negative feedback loop that regulates its own expression [30] as well as the activity of calcineurin [5, 10, 15]. Low or moderate levels of RCAN1 upregulate and high levels of RCAN1 downregulate calcineurin signaling suggesting that RCAN1 oscillates between stimulatory and inhibitory forms depending on its concentration [14, 29, 31]. The functional role of RCAN1 may change in a dose-dependent fashion but in the opposite direction to the aforementioned studies. It has been reported that RCAN1 had an inhibitory effect at low levels but an activating effect at high levels [32]. The biological activity of* rcan1* appears to be highly cell and context dependent [5]. The functional RCAN1 characteristics may be responsible for the seemingly conflicting results of the present and previous studies on the role of RCAN1 in periodontal tissues [20]. In the present study, the staining densities were found to be much lower in chronically inflamed compared to healthy periodontal tissues. However, a previous in vivo study has shown that the expression of* rcan1* is higher in treated compared to healthy periodontal tissues [20]. Based on these results one may speculate that* rcan1* is downregulated during chronic infection thus fostering the immune response and upregulated during tissue regeneration to limit the inflammatory response that may interfere with the tissue repair. Although this interpretation is vaguely defined and needs to be substantiated by additional studies, the data of the above-mentioned studies indicate that the expression of RCAN1 is at least differentially regulated in healthy as well as in untreated and periodontally affected sites, respectively.

*Rcan1* expression is mediated through numerous stimuli including calcium-elevating agents and cell receptor agonists like VEGF and thrombin [33]. Moreover, expression of* rcan1* can be induced by Gram-positive and Gram-negative bacteria [34]. The data of the present study show that the Gram-negative putative periodontal pathogen* P. gingivalis* increases the expression of* rcan1* as much as VEGF, which is one of the main regulators of* rcan1*. This and other results indicate that TLR4 receptors may be involved in the regulation of* rcan1* [34]. Furthermore, the observed phenomenon might display a short-term mechanism that limits the early immune response towards* P. gingivalis*, thus avoiding any collateral damage due to an overreacting inflammation.

Upon stimulation with VEGF and different* P. gingivalis* strains the expression of* cox2* followed almost the same pattern compared to that of* rcan1*, indicating a common mechanism that controls the expression of both genes. This finding is supported by other studies demonstrating that* cox2* and* rcan1.4* are both upregulated by calcineurin-dependent calcium signals [35, 36]. Mobilization of intracellular calcium has been described to strongly augment the promoter activity and mRNA and protein expression of* rcan1.4* and* cox2* [35]. For both genes, the calcium signal component has been further found to be dependent on calcineurin and is replicated by exogenous expression of a constitutively active NFAT, strongly suggesting that the calcium-induced gene activation is mediated by NFAT [35, 37].

It is presently thought that RCAN1 regulation of calcineurin activity can be exploited to treat various inflammatory diseases. With regard to periodontal inflammation it is noteworthy that tacrolimus, a synthetic calcineurin inhibitor like RCAN1, has been found to exert protective effects on periodontal disease progression [38, 39] indicating that “fine tuning” of the NFAT-RCAN1 negative feedback loops may modulate inflammatory process in periodontitis.

In conclusion, the data of the present study provide the first further evidence that RCAN1 may be involved in modulating inflammation in a dependence of inflammation stages manner and homeostasis in periodontal ligament. However, additional studies in an animal model of periodontitis, for example, RCAN1 knockout mice, are needed to substantiate the importance of RCAN1 in periodontal disease.
